# First Case of Complete Bladder Duplication in the Coronal Plane with Concomitant Duplication of the Urethra in an Adult Male

**DOI:** 10.1155/2013/638125

**Published:** 2013-07-17

**Authors:** Nikolaos Karpathakis, Georgia Vasileiou, Konstantinos Fasoulakis, Ioannis Heretis

**Affiliations:** Department of Urology, University Hospital of Heraklion, Voutes 71110, Heraklion, Crete, Greece

## Abstract

Duplication of the lower urinary tract is a very rare congenital anomaly which is diagnosed either at birth or during early childhood. These rare malformations are most of the times accompanied by other concomitant anomalies and are therefore diagnosed immediately after birth. In some even rarer cases there are no concomitant anomalies and symptoms thus leading to a diagnosis later in childhood. This is the first case in the literature of complete bladder duplication in the coronal plane with concomitant duplication of the urethra and no other associated anomalies in a 52-year-old male who remained asymptomatic and therefore undiagnosed for more than 5 decades.

## 1. Case Presentation

A 52-year-old male was admitted to the urology department due to sudden purulent urethral secretion. He reported a painless progressively developing palpable mass anterior to the pubic symphysis which he first noticed 18 months prior to his admittance. He also reported that the mass had subsided after the appearance of the purulent exudate. The patient had no lower urinary tract symptoms. Clinical examination revealed a palpable painless mass anterior to the pubic symphysis, and after close inspection a second epispadic urethral meatus was observed. The patient was nonfebrile with normal blood count and biochemistry. The urine culture was sterile.

**Figure 1:**
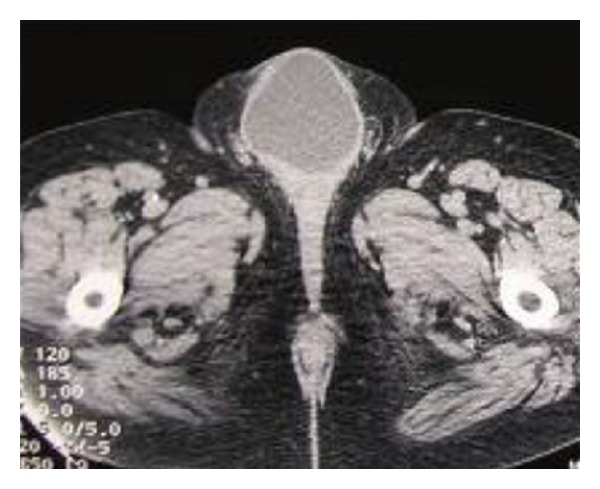
CT of the lower abdomen and pelvis showing a large cystic mass.

**Figure 2:**
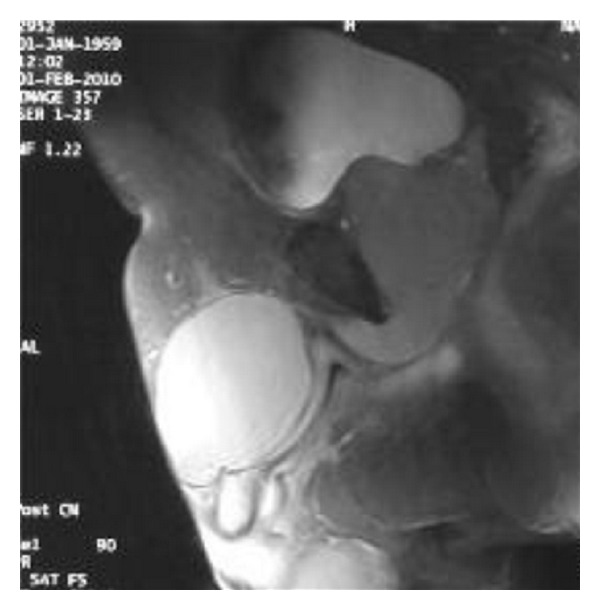
MRI showing the accessory bladder in front of the normal bladder.

A contrast-enhanced CT was performed that revealed a low-density 7 cm wide cystic mass anterior to the pubic symphysis, in the anatomic region of the suspensory ligament of the penis and in direct contact with the bladder (Figure 1). Consistency with the prostatic and membranous urethra was also reported. An MRI of the pelvis showed a large, cystic mass. Its distal part was located in the soft tissue, frontal to the pubic symphysis, and extended to the dorsal surface of the cavernous body of the penis. The central part was located in the abdomen in front of the bladder and extended through the urogenital diaphragm reaching the distal part and communicating with it through a narrow neck. The prostate was dispensed dorsally by the mass (Figure 2).

**Figure 3:**
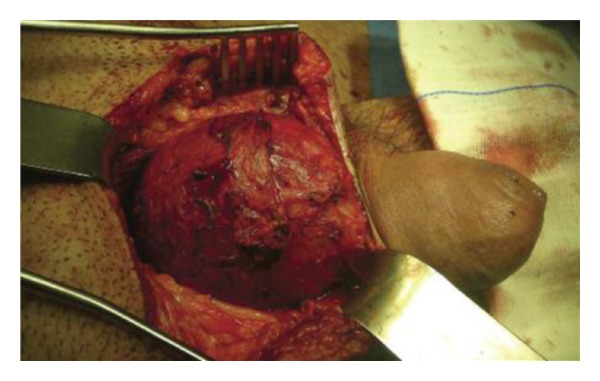
The upper part of the accessory bladder intraoperatively.

**Figure 4:**
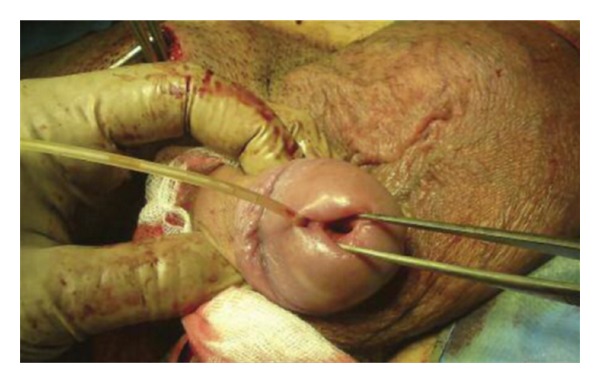
Normal urethral meatus and the epispadic meatus of the accessory urethra with a Nelaton catheter inside.

**Figure 5:**
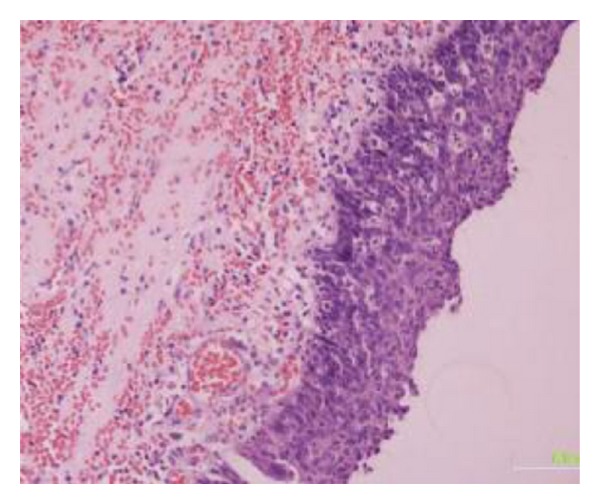
Microscopic examination of the specimen showing transitional cell epithelium followed by a fibromuscular layer.

The patient underwent an operation for removal of the mass. In front of the bladder and in direct contact with it, a large cystic mass was found (Figure 3). The mass was unrelated to the upper urinary system, and after gross inspection there was no communication with the normal bladder. The purulent content of the cystic mass drained through a complete supernumerary urethra with an epispadic urethral meatus (Figure 4). The accessory bladder was emptied and then removed leaving the normal bladder untouched. The patient was discharged five days after the surgery. The pathology report of the specimen confirmed the diagnosis of accessory bladder with transitional cell epithelium followed by a fibromuscular layer (Figure 5).

## 2. Discussion

The duplication of the lower urinary tract refers to the duplication of the bladder or/and the urethra. Abrahamson was the first to suggest a classification for bladder duplication [1]. He classified these congenital anomalies to either complete duplication of the bladder with two bladders and two separate urethras or incomplete duplication with two bladders and a common urethra. In order to separate true bladder duplication from other anomalies such as bladder diverticula or multilocular bladders, the septum between the two bladders must include muscular tissue. Abrahamson also offered two possible explanations for the embryologic origin of bladder duplication. The first one suggests that the malformation is the result of excessive constriction between the urogenital and vesicourethral portions of the ventral cloaca. The second theory suggests that a supernumerary cloacal septum indents the epithelial wall of the bladder causing it to split.

Depending on the position of the accessory bladder in relation to the normal bladder, bladder duplication is also classified as sagittal or coronal. Bladder duplication in the sagittal plane is more common with the two bladders side by side, each receiving urine from one ureter [2]. Duplication in the coronal plane is less often. The accessory bladder usually lies anterior and superior of the normal bladder [2].

Our case has several characteristics that make it unique. To our knowledge this is the 6th case of complete bladder duplication in the coronal plane with no other associated anomalies reported in literature. These cases are so uncommon because usually such malformations are accompanied by other congenital anomalies, urologic or not. Due to the common embryologic origin of lower urinary tract and lower intestines, gastrointestinal anomalies such as lower GI duplication, anorectal atresia, imperforate anus, and others are common. Other anomalies of the genital system are also present in numerous similar cases such as duplication of external genitalia like diphallus and duplication of the glans or bifid scrotum. Finally there are reports of several cases with concomitant vertebral anomalies like duplicate spine and spina bifida. In a review of all cases with bladder duplication (sagittal or coronal) by Coker et al. [3], only 6 out of 39 cases were completely free of other congenital anomalies. Four out of these six reports referred to duplications in the coronal plane [4–7]. In 2012 a fifth case of a completely asymptomatic bladder duplication in the coronal plane with no other concomitant anomalies was presented by Pirincci et al. [8]. Complete absence of any symptoms and other anomalies led the parents and the medical team to decide against any intervention.

The most unique characteristic of our case is the age of our patient. The supernumerary bladder remained asymptomatic for more than 5 decades until an infection, probably due to the bladders' colonization from bacteria through the epispadic urethra, occurred and led to symptomatic disease. Another factor that contributed to the late diagnosis was the fact that the epispadic urethral meatus was very close to the normal meatus thus making it almost impossible to see. This proves that if left untreated at early childhood, this type of malformation can remain asymptomatic for many decades although there is always the possibility of becoming symptomatic probably due to infection.

## References

[1] Abrahamson J (1961). Double bladder and related anomalies: clinical and embryological aspects and a case report. *British Journal of Urology*.

[2] Berrocal T, Novak S, Arjonilla A, Gutiérrez J, Prieto C, Urrutia MJ (1999). Complete duplication of bladder and urethra in the coronal plane in a girl: case report and review of the literature. *Pediatric Radiology*.

[3] Coker AM, Allshouse MJ, Koyle MA (2008). Complete duplication of bladder and urethra in a sagittal plane in a male infant: case report and literature review. *Journal of Pediatric Urology*.

[4] Bae KS, Jeon SH, Lee S-J (2005). Complete duplication of bladder and urethra in coronal plane with no other anomalies: case report with review of the literature. *Urology*.

[5] Cheng EY, Maizels M (1996). Complete duplication of the bladder and urethra in the coronal plane: case report. *Journal of Urology*.

[6] Oğuzkurt P, Ozalevli SS, Alkan M, Kayaselcuk F, Hiçsönmez A (2006). Unusual case of bladder duplication: complete duplication in coronal plane with single urethra and no associated anomalies. *Urology*.

[7] Singh JP, Mehra S, Nagabhushanam V (1973). Complete duplication of bladder and urethra: a case report with review of the literature. *Journal of Urology*.

[8] Pirincci N, Gecit I, Gunes M, Tanik S, Ceylan K (2013). Complete duplication of the bladder and urethra in the coronal plane: case report with review of the literature. *Urologia Internationalis*.

